# Normal Bone Mineral Density Associates with Duodenal Mucosa Healing in Adult Patients with Celiac Disease on a Gluten-Free Diet

**DOI:** 10.3390/nu9020098

**Published:** 2017-01-31

**Authors:** Tiziana Larussa, Evelina Suraci, Maria Imeneo, Raffaella Marasco, Francesco Luzza

**Affiliations:** Department of Health Sciences, University of Catanzaro “Magna Graecia”, Viale Europa, Germaneto, Catanzaro 88100, Italy; tiziana.larussa@gmail.com (T.L.); e.suraci@libero.it (E.S.); graziaimeneo@hotmail.it (M.I.); raffaellamarasco1@gmail.com (R.M.)

**Keywords:** celiac disease, bone disorders, osteoporosis, histopathology, intestinal mucosa healing

## Abstract

Impairment of bone mineral density (BMD) is frequent in celiac disease (CD) patients on a gluten-free diet (GFD). The normalization of intestinal mucosa is still difficult to predict. We aim to investigate the relationship between BMD and duodenal mucosa healing (DMH) in CD patients on a GFD. Sixty-four consecutive CD patients on a GFD were recruited. After a median period of a 6-year GFD (range 2–33 years), patients underwent repeat duodenal biopsy and dual-energy X-ray absorptiometry (DXA) scan. Twenty-four patients (38%) displayed normal and 40 (62%) low BMD, 47 (73%) DMH, and 17 (27%) duodenal mucosa lesions. All patients but one with normal BMD (23 of 24, 96%) showed DMH, while, among those with low BMD, 24 (60%) did and 16 (40%) did not. At multivariate analysis, being older (odds ratio (OR) 1.1, 95% confidence interval (CI) 1.03–1.18) and having diagnosis at an older age (OR 1.09, 95% CI 1.03–1.16) were associated with low BMD; in turn, having normal BMD was the only variable independently associated with DMH (OR 17.5, 95% CI 1.6–192). In older CD patients and with late onset disease, BMD recovery is not guaranteed, despite a GFD. A normal DXA scan identified CD patients with DMH; thus, it is a potential tool in planning endoscopic resampling.

## 1. Introduction

Celiac disease (CD) is a chronic autoimmune disorder occurring in genetically predisposed individuals, triggered by gluten and related prolamins contained in wheat, barley, and rye. The resulting malabsorption due to small intestinal injury leads to systemic damage, mostly related to nutritional deficiencies [1]. Serological screening tests are available to select individuals needing to undergo diagnostic endoscopic biopsy of the duodenal mucosa. They are immunoglobulin (Ig)A anti-tissue transglutaminase (tTG) and anti-endomysium antibodies-IgA (EMA), both showing a specificity close to 100% and a sensitivity greater than 90% [2]. Serology is also a useful tool in monitoring adherence and response to a gluten-free diet (GFD), although it may not be representative of a complete recovery of the intestinal mucosa [3].

Reduced bone mineral density (BMD) is found in more than 50% of newly diagnosed patients with CD, possibly due to impaired calcium and vitamin D absorption [4]. Besides micronutrient malabsorption, it is conceivable that chronic inflammation can predispose CD patients, whether on a GFD or not, to mineral metabolism derangement [5]. Indeed, a lack of calbindin and calcium-binding protein, the vitamin D-regulated protein implicated in calcium uptake from the intestinal lumen, has been described in the areas of damaged mucosa [6]. Hyperparathyroidism sustained by a chronic inflammatory state is another implicated factor, since high parathormone (PTH) values are frequent in CD patients even in the presence of normal circulating vitamin D levels [7]. Release of proinflammatory cytokines such as interleukin (IL)-6, IL-1β, tumor necrosis factor (TNF)-*α*, and interferon (IFN)-*γ* has been implicated in bone remodeling during CD, as well as the receptor activator of nuclear factor kappaB (RANK)/RANK ligand (RANKL)/osteoprotegerin (OPG) axis, according to which the reabsorbing activity performed by RANKL is counteracted by the effects of its natural decoy receptor OPG [8]. A lower OPG/RANKL ratio was found in CD patients with recovery of intestinal mucosa and positively correlated with a reduced BMD [9]. A strict GFD restores mucosal damage and reverses the biochemical evidence of calcium malabsorption, resulting in normal BMD in these treated patients [10]. Nevertheless, a long-term impairment of bone mineralization can persist in some otherwise healthy CD patients adhering to a GFD and harboring negative serology [11]. In this regard, it is proper to recall that naturally gluten-free products are often low in B vitamins, calcium, vitamin D, iron, zinc, magnesium, and fiber, while enrichment of gluten-free products is not so common. Therefore, dietary advice other than gluten withdrawal seems to be necessary in CD patients in order to better choose the composition of foods and prevent complications due to malnutrition [12]. At the same time, incomplete mucosal recovery represents a challenge for clinicians, since it can occur in apparently asymptomatic CD patients despite adequate GFD and negative serology [13]. These findings suggest the importance of a follow-up biopsy after CD diagnosis and the need for parameters other than serology or dietary assessment to target the optimal timing of the endoscopic repeat procedure.

This study aimed at investigating the relationship between BMD assessment and mucosal duodenal status in adult CD patients on a GFD.

## 2. Materials and Methods

### 2.1. Patient Recruitment and Study Design

Between January 2012 and September 2015, 64 consecutive asymptomatic outpatients with CD (18 male and 46 female; median age 36 years, range 18–69) were selected for the study. Patients had to be adherent to a GFD for at least 2 years, harbored persistent (at least 18 months) negative CD-related serology, and reported no current gastrointestinal symptoms. Diagnosis of CD needed to be performed on the basis of clinical presentation, positive CD-related serology, and suggestive histological findings on duodenal biopsy [14]. All patients had atrophic disease at diagnosis and did not repeat biopsy before recruitment, neither a baseline BMD measurement was performed except for a subgroup of 25 patients. Exclusion criteria included pregnancy, breast-feeding, and a previous diagnosis of hematological diseases or hormonal and metabolic disorders which could account for low BMD. Data on height, weight, time since diagnosis, onset of symptoms, clinical presentation, age at menarche, cycle regularity, menopausal status, drug use, calcium intake, life style (such as levels of physical activity), smoking, history of fracture, and other relevant co-morbidities were collected. Each patient, in a time frame of 6 months from recruitment, was submitted to gastroscopy with duodenal resampling along with measurement of BMD and serological assay.

### 2.2. Dietary Assessment and Body Mass Index Calculation

Clinicians assessed dietary compliance by periodic interview during follow-up visits in order to demonstrate deliberate or inadvertent gluten intake. Adherence to a GFD was classified as good according to Leffler et al. in a standardized fashion by analysis of a 3-day food record [15]. The body mass index (BMI) was calculated as weight in kilograms divided by the square of height in meters (normal range 18–24.9 kg/m^2^).

### 2.3. Serology and Laboratory Parameters

Blood samples were collected in the morning after a 12 h fast in order to measure serum levels of calcium, vitamin D, and PTH. Patients were tested for tTG and EMA antibodies (both IgA and IgG classes). Serum tTG antibodies were investigated by an enzyme-linked immunosorbent assay (ELISA; A. Menarini diagnostics, Florence, Italy), a unit value of ≥5 being positive. Serum EMA was determined using an indirect immunofluorescence method with a monkey distal esophagus as a substrate; a dilution of 1:5 was considered positive (A. Menarini diagnostics).

### 2.4. Duodenal Mucosa Sampling and Histology

Patients underwent repeat duodenal biopsy after a period of at least 2 years since the beginning of the GFD (median 6 years, range 2–33). At least four biopsy specimens were collected from the distal duodenum during upper gastrointestinal endoscopy, fixed in 10% formalin and paraffin-embedded. Intraepithelial lymphocytes have been identified using CD3 immunostaining, and a value of ≤25 lymphocytes/100 epithelial cells was considered normal. Histological changes were classified according to Marsh criteria (Stage 0: normal mucosa; Stage 1: increased number of intraepithelial lymphocytes; Stage 2: crypts proliferation; Stages 3a-3b-3c: respectively mild, moderate, and severe villous atrophy) [16]. Marsh Stage 0 at repeat biopsy was considered duodenal mucosa healing (DMH). All evaluations were carried out in a blinded fashion by the same pathologist without prior knowledge of patient history.

### 2.5. Measurement of Bone Mineral Density

At the time of duodenal resampling, BMD in the lumbar spine and femoral neck was measured by means of dual-energy X-ray absorptiometry (DXA) according to standard procedures. Values were expressed as standard deviation scores, which compare individual BMD determinations to those of young adults (T-score). Based on the World Health Organization criteria, patients with a T-score (in the lumbar spine or femoral neck or both) between −2.5 and −1 were considered osteopenic, while a T-score < −2.5 identified osteoporosis [17].

### 2.6. Ethical Considerations

The study was carried out according to the Declaration of Helsinki. The patients received oral and written information about the study. All participators were informed that participation was voluntarily and that they could withdraw at any time without consequences. The study protocol was approved by the local research Ethical Committee, and written informed consent was obtained from all the participants.

### 2.7. Statistics

At univariate analysis, BMD and duodenal mucosa status were analyzed in relation to all the considered variables by means of an unpaired Student’s *t*-test, a chi-square test, or a Fisher’s exact test, as required. A difference was considered significant if the *p*-value was less than 0.05. The odds ratio (OR) of having low BMD and DMH, given the presence of a particular variable, was used as a measure of association and adjusted for the effect of confounding variables by multivariate logistic regression analysis (SPSS Statistic 16.0; IBM, Armonk, NY, USA).

## 3. Results

### 3.1. Clinical Findings and Serology

Table 1 shows the demographic and clinical characteristics of the participants. No patient had a history of bone fractures or of any endocrine, kidney, or liver disorder accountable for bone derangement. Patients did not receive supplementation with vitamins, calcium, or iron, nor had they taken medications capable of acting on bone metabolism, such as steroids. The three patients on menopausal status did not assume hormone replacement therapy for their condition. Intestinal resections or small bowel diseases causing malabsorption were absent. All patients showed a good adherence to a GFD. No special diet regimen was found (such as vegetarian or vegan diet), nor were excess or deficiency levels of physical activity worth noting. Serum calcium, vitamin D, and parathyroid hormone levels were normal, and both anti-tTG and EMA were negative in all patients.

### 3.2. BMD and Histology

Forty (62%) of the 64 CD patients displayed low BMD, with 2 (5%) accounting for osteoporosis and 38 (95%) for osteopenia. BMD was normal in the remaining 24 patients (38%). With the exception of age at evaluation and diagnosis, and duration of the GFD, characteristics of patients did not differ between subjects with normal and low BMD at univariate analysis (Table 2). However, only being older and having CD diagnosis at an older age remained independently associated with low BMD at multivariate analysis (Table 2). DMH was found in 47 (73%) patients, while 17 (27%) showed duodenal mucosa lesions (*n* = 9, Marsh Stage 1; *n* = 2, Marsh Stage 2; and *n* = 6, Marsh Stage 3). All patients but one with normal BMD (*n* = 23, 96%) showed DMH. Among patients with low BMD, 24 (60%) showed DMH, while 16 (40%) did not (Table 3). Characteristics of the 64 CD patients on a GFD according to duodenal mucosa status are summarized in Table 3. Even though age, age at diagnosis, and BMI closely approached statistical significance, at multivariate analysis, a normal BMD was the only variable independently associated with DMH (OR 17.5, 95% CI 1.6–192).

## 4. Discussion

In spite of long-term strict adherence to a GFD and persistent negative CD-related serology, a high prevalence of low BMD (62%) has been shown in CD patients of this study. These findings suggest that risk factors other than villous atrophy are possibly involved in bone injury, such as diagnosis of CD in adult life, irregular adherence to a GFD, lactose intolerance, and nutritional deficiency related to naturally gluten-free foods or to the composition of gluten-free products.

At the same time, a significant proportion of patients (27%) displayed duodenal mucosa lesions, and they all had a low BMD. It has been already observed that DMH after a GFD is not achieved in a considerable proportion of CD patients, notwithstanding prolonged and strict adherence to the diet [3]. Pekki et al. found, both at diagnosis and after one year GFD, a relationship between an impaired T-score and duodenal mucosa lesions as verified by follow-up biopsies [18]. Therefore, even in the presence of a negative serology and lack of intestinal symptoms, a low BMD could be taken into account when considering a persistent duodenal mucosa lesion. As an additional value, this study focuses on the fact that a normal BMD predicts DMH, since all but one patient who displayed a normal BMD showed DMH (96%) and independently associated with DMH (OR 17.5).

Compared to patients with abnormal DXA findings, patients with both normal BMD and DMH were younger, had an earlier diagnosis, and a longer period of GFD. Nevertheless, adjustment of variables with each other no longer confirmed that age, age at diagnosis, and duration of the GFD were independently associated with duodenal mucosa status at multivariate analysis—only a normal BMD has this association. It is remarkable that, in the same series, negative CD-related serology was associated in only 73% of patients who showed DMH. Even though the presence of non-atrophic lesions of the intestinal mucosa (i.e., Marsh Stages 1 and 2) cannot be considered sufficient to establish the diagnosis of CD, they should be regarded as a lack of histological recovery after a long-term strict adherence to a GFD [19,20]. Accordingly, we established that DMH corresponds to Marsh Stage 0.

An impaired bone mineralization is a frequent finding during CD, at both diagnosis and after a GFD, widely ranging from 38% to 72% and 9% to 47%, respectively [21]. In the subgroup of 25 patients who performed DXA at the time of CD diagnosis, 22 (88%) had an impaired bone mineralization (data not shown), and this may explain the high proportion of low BMD (62%) still found after a GFD in all the study patients.

In untreated CD patients, calcium malabsorption is due mechanically to intestinal mucosal damage and functionally to the presence of intraluminal unabsorbed fatty acids which bind calcium in the intestinal lumen and may reduce dietary vitamin D absorption [22]. In our series, it was confirmed that no patient displayed abnormal levels of circulating calcium, vitamin D, and PTH [23]. Even though serum calcium levels may not adequately reflect calcium absorption, we did not search for bone resorption markers since they are rarely used in clinical practice, while being an accurate method of bone health assessment [24]. Moreover, chronic release of proinflammatory cytokines and other factors of bone remodeling, such as estrogens, androgens, insuline-like growth factor-1, and PTH contribute to low BMD in CD patients and they are still under investigation [25]. All the above conditions are reversed by a GFD, which repairs mucosal damage. Nevertheless, diet is found to improve but often not to normalize BMD, suggesting that further strategies are needed to manage bone derangement in CD patients [26]. Current data did not support evidence for additional benefits derived from dietary supplementation (e.g., with calcium and vitamin D) in adult CD patients. However, in some special situations, such as osteoporosis detected in celiac postmenopausal women, it could be useful to begin treatment with hormone replacement therapy or bisphosphonates. In addition, education on the importance of lifestyle changes, such as regular exercise, smoking cessation, and excessive alcohol intake, should be provided [27].

Many risk factors for derangement of bone mineralization and its relationship with the duodenal mucosa status have been here assessed in a homogeneous group of CD patients on a GFD. Indeed, age at evaluation and diagnosis, and the duration of the GFD, were significantly different between patients with low and normal BMD, while no other differences in acknowledged risk factors were found between the two groups, nor the clinical presentation of CD. This means that older patients, diagnosed at a later age and with a shorter GFD period, are at higher risk of bone derangement. Studies on pediatric CD population support these findings, as BMD normalization is achieved in young CD patients initiating a GFD early [28]. Furthermore, adjustment of variables with each other indicates only age and age at diagnosis as independent risk factors associated with BMD. Consequently, the duration of the GFD needed to normalize BMD remains unclear [29]. Given that CD is a risk factor for bone health impairment and that GFD alone is not enough to restore BMD, efforts should be focused to identify predicting factors for bone demineralization in CD patients.

The novel finding of this study is that DXA may have a place in the follow-up of CD patients, particularly to help selecting patients who need a control biopsy and those who did not. Indeed, the association between normal BMD and DMH excludes patients with a normal DXA from endoscopic biopsy resampling, shifting the attention on CD patients who display an abnormal DXA and, possibly, harbor duodenal mucosa lesions.

Data on the correlation between bone derangement and Marsh Stage in newly diagnosed CD patients have been produced, and this supports the role of malabsorption in determining low BMD in untreated individuals [30]. However, little is known about the mucosal intestinal recovery and the causes of its delay. To date, the time for scheduling repeat biopsies is still debated and no agreement exists on the best time to plan biopsy follow-up [31]. If a normal BMD will be confirmed to associate with DMH in CD patients on a GFD, the use of DXA could be proposed as an adjunctive tool in the management of CD patients during the follow-up.

## 5. Conclusions

This study confirms that BMD derangement and incomplete DMH are frequent findings in adult CD patients on a GFD. Furthermore, the novel finding that a normal BMD associates with DMH suggests that DXA may be a useful tool in the management of adult CD patients and the timely planning of endoscopic biopsy resampling.

## Figures and Tables

**Table 1:** Characteristics of the 64 patients with celiac disease on a gluten-free diet.

Variables	Values
Sex	18 (28) males; 46 (72) females
Age, years	36.1 ± 10.7 (range 18–69)
Age at diagnosis, years	28 ± 14.3 (range 2–64)
Duration of GFD, years	8.3 ± 6.6 (range 2–33)
BMI (Kg/m^2^)	21.9 ± 1.8 (range 19.1–25.6)
Clinical presentation	
Malabsorption	36 (56)
Diarrhea	16 (25)
Dyspepsia	7 (11)
Extraintestinal symptoms	2 (3)
Screen-detected	3 (5)
Smoke	19 (29)
Menopausal status	3 (7)
History of fracture	0 (0)

BMI = body mass index; GFD = gluten-free diet; SD = standard deviation. Values are numbers (%) or means ± SD as indicated.

**Table 2:** Characteristics of the 64 patients with celiac disease on a gluten-free diet according to bone mineral density as assessed by dual energy X ray absorptiometry.

Variables	Normal BMD *n* = 24	Low BMD *n* = 40	*p*	OR (95% CI) Adjusted ^a^	*p*
Sex					
Male	7 (29)	11 (28)	0.83	1.56 (0.41–5.86)	0.51
Female	17 (71)	29 (72)			
Age, years	30.1 ± 10.6	39.6 ± 9.5	0.0002	1.1 (1.03–1.18)	0.004
Age at diagnosis *, years	20.1 ± 14.9	37.7 ± 11.9	0.0002	-	-
BMI (Kg/m^2^)	22.3 ± 1.7	21.7 ± 1.8	0.08	0.8 (0.6–1.17)	0.31
Smoke	7 (29)	12 (30)	0.97	0.86 (0.23–3.15)	0.82
Clinical presentation ^b^					
Malabsorption	12 (50)	24 (60)	0.34	0.83 (0.18–3.66)	0.81
Diarrhea	7 (29)	9 (23)	0.48		
Dyspepsia	3 (13)	4 (10)	0.71		
Extraintestinal symptoms	0 (0)	2 (4)	0.27		
Screen-detected	2 (8)	1 (3)	0.26		
Duration of GFD ^c^, years	10.1 ± 7.6	7.2 ± 5.7	0.04	0.65 (0.19–2.18)	0.48

CI = confidence interval; MVA = multivariate analysis; OR = odds ratio; SD = standard deviation; BMD = bone mineral density; BMI = body mass index; GFD = gluten-free diet. Values are numbers (%) or means ± SD as indicated. Means were compared with the use of a Student’s *t*-test and proportions with the use of a chi-square test or Fisher’s exact test. OR with 95% CI in brackets is given. * Due to collinearity with age, this variable entered a separate MVA with all other variables than age and leads to OR 1.09, 95% CI 1.03–1.16, *p* = 0.003, while the other variables did not reach the statistical significance (*p* > 0.05). ^a^ All variables except age at diagnosis (due to collinearity with age) entered MVA. Male gender, smoking, conventional clinical presentation, and patients with GFD ≤ 7 as references in MVA. ^b^ Clinical presentation entered MVA analysis as categorical variable, assuming malabsorption and diarrhea as conventional presentation and screen-detected, extraintestinal symptoms and dyspepsia as unusual presentation. ^c^ Duration of GFD entered MVA as categorical variable, subgrouping subjects with GFD ≤ 7 (*n* = 36) and those with GFD ≥ 8 (*n* = 28).

**Table 3:** Characteristics of the 64 patients with celiac disease on a gluten-free diet according to duodenal mucosa healing as assessed by Marsh classification.

Variables	Mucosal Healing *n* = 47	Mucosal Lesions *n* = 17	*p*	OR (95% CI) Adjusted ^a^	*p*
Bone mineral density					
Normal	23	1	0.001	17.5 (1.6–192)	0.019
Low	24	16			
Sex					
Male	12	6	0.53	0.5 (0.11–2.52)	0.43
Female	35	11			
Age, years	35.1 ± 10.5	39.8 ± 11.3	0.06	1.1 (0.9–1.34)	0.34
Age at diagnosis, years	26.4 ± 14	32.5 ± 14.7	0.07	0.9 (0.76–1.08)	0.26
BMI (Kg/m^2^)	22.1 ± 1.8	21.4 ± 1.7	0.08	0.9 (0.64–1.48)	0.91
Smoke	13	6	0.55	2.7 (0.56–13.5)	0.20
Clinical presentation ^b^					
Malabsorption	28 (60)	8 (47)	0.4	1.3 (0.23–7.6)	0.75
Diarrhea	10 (21)	6 (35)	0.32		
Dyspepsia	6 (13)	1 (6)	0.66		
Extraintestinal symptoms	0 (0)	2 (12)	0.06		
Screen-detected	3 (6)	0 (0)	0.55		
Duration of GFD ^c^, years	8.7 ± 6.4	7.3 ± 7.6	0.23	0.17 (0.01–1.65)	0.12

CI = confidence interval; MVA = multivariate analysis; OR = odds ratio; SD = standard deviation; BMI = body mass index; GFD = gluten-free diet. Values are numbers (%) or means ± SD as indicated. Means were compared with the use of a Student’s *t*-test and proportions with the use of a chi-square test or Fisher’s exact test. OR with 95% CI in brackets is given. ^a^ All variables entered MVA. Male gender, smoking, conventional clinical presentation, and patients with GFD ≤ 7 as references in MVA. ^b^ Clinical presentation entered MVA analysis as categorical variable, assuming malabsorption and diarrhea as conventional presentation and screen-detected, extraintestinal symptoms and dyspepsia as unusual presentation. ^c^ Duration of GFD entered MVA as categorical variable, subgrouping subjects with GFD ≤ 7 (*n* = 36) and those with GFD ≥ 8 (*n* = 28).
